# Dietary quality in a sample of adults with type 2 diabetes mellitus in Ireland; a cross-sectional case control study

**DOI:** 10.1186/1475-2891-12-110

**Published:** 2013-08-06

**Authors:** Alison E Murray, Aoibheann M McMorrow, Eamonn O’Connor, Catherine Kiely, Oscar Mac Ananey, Donal O'Shea, Mikel Egaña, Fiona E Lithander

**Affiliations:** 1 Department of Clinical Medicine, Trinity College Dublin, Trinity Centre for Health Sciences, St James’s Hospital, Dublin 8, Ireland; 2 Department of Physiology, Trinity College Dublin, Biomedical Sciences Institute, Pearse St, Dublin 2, Ireland; 3 Department of Endocrinology, St Vincent's University Hospital, University College Dublin, Dublin 4, Ireland; 4 ANU Medical School and The John Curtin School of Medical Research, The Australian National University, Canberra ACT 0200, Australia

**Keywords:** Dietary quality, Nutrient intake, Type 2 diabetes mellitus

## Abstract

**Background:**

A number of dietary quality indices (DQIs) have been developed to assess the quality of dietary intake. Analysis of the intake of individual nutrients does not reflect the complexity of dietary behaviours and their association with health and disease. The aim of this study was to determine the dietary quality of individuals with type 2 diabetes mellitus (T2DM) using a variety of validated DQIs.

**Methods:**

In this cross-sectional analysis of 111 Caucasian adults, 65 cases with T2DM were recruited from the Diabetes Day Care Services of St. Columcille’s and St. Vincent’s Hospitals, Dublin, Ireland. Forty-six controls did not have T2DM and were recruited from the general population. Data from 3-day estimated diet diaries were used to calculate 4 DQIs.

**Results:**

Participants with T2DM had a significantly lower score for consumption of a Mediterranean dietary pattern compared to the control group, measured using the Mediterranean Diet Score (Range 0–9) and the Alternate Mediterranean Diet Score (Range 0–9) (mean ± SD) (3.4 ± 1.3 vs 4.8 ± 1.8, *P* < 0.001 and 3.3 ± 1.5 vs 4.2 ± 1.8, *P* = 0.02 respectively). Participants with T2DM also had lower dietary quality than the control population as assessed by the Healthy Diet Indicator (Range 0–9) (T2DM; 2.6 ± 2.3, control; 3.3 ± 1.1, *P* = 0.001). No differences between the two groups were found when dietary quality was assessed using the Alternate Healthy Eating Index. Micronutrient intake was assessed using the Micronutrient Adequacy Score (Range 0–8) and participants with T2DM had a significantly lower score than the control group (T2DM; 1.6 ± 1.4, control; 2.3 ± 1.4, *P* = 0.009). When individual nutrient intakes were assessed, no significant differences were observed in macronutrient intake.

**Conclusion:**

Overall, these findings demonstrate that T2DM was associated with a lower score when dietary quality was assessed using a number of validated indices.

## Introduction

Dietary intake plays a role in both the aetiology and management of type 2 diabetes mellitus (T2DM), and is a key modifiable risk factor [1]. Dietary intake, characterised by a high intake of energy and nutrients such as fat and sugar accompanied by a low intake of fibre, has been shown to increase the risk of T2DM [2]. Many national and international associations have produced evidence-based dietary recommendations, including the European Association for the Study of Diabetes, the American Diabetes Association (ADA) and the Canadian Diabetes Association (CDA), in order to help individuals with T2DM to achieve a dietary intake that is not only nutritionally adequate but can also help to optimise their metabolic control. However, research has shown that individuals with T2DM fail to achieve such nutrient recommendations [3-5].

Recent evidence suggests that using dietary patterns to model dietary intake as opposed to nutrient specific based approaches may offer greater health benefits, as dietary patterns are suggested to offer a broader representation of dietary intake and have been shown to mediate effects on health and disease risk [6,7]. Dietary patterns take into account the synergistic effects of whole foods and may prove more beneficial in terms of assessing the quality of dietary intake [8]. For instance, the synergistic effects of the food groups within a Mediterranean dietary pattern were supported by analyses by Trichopoulou and colleagues, who found that dietary pattern as a whole was inversely associated with mortality [9].

A number of validated dietary quality indices (DQIs) have been developed to assess the quality of dietary intake by using dietary pattern analyses. Such validated DQIs include the Alternate Healthy Eating Index [10], the Mediterranean Diet Score [9,11], the Alternate Mediterranean Diet Score [12] and the Healthy Diet Indicator [13]. There is limited data available on the use of such DQIs in populations with T2DM.

The aim of this cross-sectional study was to measure the dietary intake of a sample of adults with T2DM and to assess the overall quality of dietary intake using a selection of validated dietary quality indices.

## Methods

### Participants

Participants with T2DM were recruited from the Diabetes Day Care Services of St. Columcille’s Hospital and St. Vincent’s Hospital, Dublin, Ireland. Participants were originally referred to this service by their general practitioner and the cohort consisted of a mixture of both new and returning patients. All participants with T2DM were required to see a dietitian as part of the service although it is unknown how many did. A control group, of similar age and physical activity level was recruited from the same catchment area as the case subjects through distribution of advertisements to local businesses. All participants provided informed written consent before participation. The study was conducted in accordance with the principles of the Declaration of Helsinki and was approved by the Trinity College Dublin Faculty of Health Sciences Research Ethics Committee.

Participants with T2DM were classified as suitable for inclusion if they were between 30 and 75 years of age, had a date of diagnosis of T2DM within the previous 10 years and had a glycosylated hemoglobin (HbA1c) level of <10%. Participants with T2DM were excluded if either persistent proteinuria (urinary protein >2 mg/dL) or excess urinary creatinine (>2 mg/dL) were detected. Diagnoses of peripheral artery disease or coronary heart disease were also grounds for exclusion. Controlled hypertensive participants were admitted to the study. Non-diabetic, healthy control participants were considered eligible for inclusion if free from any cardiovascular disease or any other serious co-morbidity assessed by medical questionnaire. Control participants received a full medical examination performed by a qualified medical practitioner prior to participation. All participants were classified as sedentary, defined as performance of less than 1 hour of moderate intensity physical activity per week during the previous six months. This measure was confirmed using the Low Level Physical Activity Recall (LOPAR) questionnaire [14] and the wearing of an RT3 accelerometer (Stayhealthy Inc, Monrovia, California) for 5 consecutive days. Data were also obtained on current medications used by all participants.

### Biochemical analyses

Blood samples were collected from participants following a 12 hour overnight fast. Samples were analysed for plasma glucose, insulin, HbA1c, total cholesterol, high density lipoprotein cholesterol, low density lipoprotein cholesterol and triacylglycerides using standardised methods. Serum insulin was not measured in the control population due to cost limitations. All biochemical analyses were conducted at the haematological laboratory of St. Columcille’s Hospital, Dublin, Ireland.

### Assessment of dietary intake

Dietary intake was assessed using an estimated 3 day diet diary (Medical Research Council, Human Nutrition Research Food Diary, Cambridge, UK). Participants were asked to record all food and beverages consumed over 3 consecutive days comprising 2 weekdays and 1 weekend day and received verbal and written instructions on how to complete the diary. For each food or beverage item, participants were asked to provide details of brand names, cooking methods used, nutritional additives used during cooking, portion size consumed and details on any leftovers. Participants were instructed to record portion sizes as household measures, such as cups and tablespoons, weights given by manufacturers on packaging or as measures of small, medium or large portion sizes with the assistance of a food atlas provided within the food diary. The food atlas provided a pictorial representation of these portion sizes for 15 commonly consumed foods. Participants were also instructed to answer a supplemental dietary questionnaire contained within the food diary which consisted of 12 questions used to acquire information on types and brands of milk, butters, spreads, oils and breads used, whether the fat and skin of meat is usually consumed and food preparation techniques used. Details of recipes used were also sought, including a list of ingredients. Participants were encouraged to keep the food diary as they went about their day and to refrain from recording retrospectively by memory. Participants were instructed to maintain usual eating habits throughout the assessment period. Data on the use of nutritional supplements were also collected and if consuming nutritional supplements, participants were asked to provide the brand name, the name of the nutritional supplement in full and the dosage of supplement taken.

Reported dietary intake was assessed for validity through application of the Goldberg equation to determine levels of mis-reporting [15]. Under-reporters were defined as those with an energy intake to basal metabolic rate (EI_rep_:BMR_est_) ratio of < 1.1.

### Nutrient analyses

Nutrient and food group analyses were conducted using WISP (Weighed Intake Software Package) version 3.0 (Tinuviel Software) nutrient analysis software programme incorporating the use of McCance and Widdowson Food Composition Tables 5^th^ and 6^th^ editions with supplements. Whilst WISP contains 17 standard food groups, the current analyses necessitated the creation of new food group variables, including wholegrains, legumes, nuts, seeds, soy, red meat, white meat and processed meat, to enable application of the chosen DQIs. This was carried out within the WISP software programme by allocation of the appropriate food code to the newly defined food groups. In addition, a Micronutrient Adequacy Score [16] ranging from 0–8 was calculated for eight micronutrients discussed in the ADA and CDA evidence based nutritional guidelines for diabetes management (vitamin C, vitamin D, vitamin E, folate, potassium, magnesium, sodium and zinc). Each micronutrient was assigned a score of 1 if the recommended daily allowance (RDA) [17] was met and a score of 0 if the RDA was not met. For sodium, an additional rule was applied wherein a score of 0 was given if intake exceeded the upper tolerable limit [18].

### Assessment of dietary quality

Dietary quality was assessed using four validated DQIs. DQIs use the data from the quantitative nutritional analysis of dietary intake in order to apply a resultant qualitative score. This score is then used to predict the health outcomes that are related to the dietary pattern consumed [19].

#### The alternate healthy eating index

This DQI is an adaptation by McCullough and colleagues (2002) of the original Healthy Eating Index. The 9 components are scored on a range of 0–10. The score for each component is proportional to the extent which the dietary guideline is met [10].

#### The healthy diet indicator

The Healthy Diet Indicator was developed based on the World Health Organisation (WHO) guidelines for diet and nutrition in the prevention of chronic disease. These guidelines were subsequently updated in 2003, however nutritional recommendations remain unchanged. This index applies a dichotomous scoring method, with a total score range of 0–9. A score of +1 is given to those who meet the recommendation for a given dietary component and a score of 0 is given to those that do not meet the recommendation [13].

#### The mediterranean diet score

Developed by Trichopoulou *et al.* (1995; 2003), this diet quality assessment tool is based on the traditional dietary pattern of the Mediterranean region. The index has a total score range of 0–9. The sex-specific median intake is calculated for each dietary constituent and is used as a cut-off value to aid application of scores. Consumption of greater than the median amount is awarded a score of +1, with the exception for meat and dairy, where consumption of greater than the median amount is awarded a score of 0 [9,11].

#### The alternate mediterranean diet score

Fung and colleagues (2005) developed this adaptation of the traditional Mediterranean Diet Score. Whilst similar to the original Mediterranean Diet Score, modifications were made to the original Mediterranean Diet Score based on dietary patterns and behaviours that were repeatedly found to be associated with reduced chronic disease risk [12].

### Anthropometry

All anthropometric measures were carried out with participants wearing light clothing and with shoes removed. Body mass was determined on a set of platform beam scales (AVERY, United Kingdom) measured to the nearest 0.1 kg. Height was measured to the nearest 0.5 cm, using a SECA™ Stadiometer (SECA Ltd., Germany). Determination of height was made whereby the participant stood with their back to the stadiometer while looking straight ahead. Height was recorded after subjects fully inhaled. Participant waist circumference (WC) was measured from the level of the umbilicus, with the hip measurement collected in line with the greater trochanter. Both measurements were determined using a tape measure, and measured to the nearest 1 mm. Waist:hip ratio (WHR) was then calculated from these measures.

### Statistical analyses

Values are expressed as means and standard deviation. Continuous variables were assessed for normality of distribution by analysing skewness, kurtosis, Shapiro-Wilk and Kolmogorov-Smirnov values and through inspection of Q-Q plots. Continuous variables that were skewed were log, square root and inverse transformed as appropriate before statistical analyses were performed. Independent sample t-tests were used to test for differences between group means of the T2DM group and controls. Mann Whitney-U tests were used to test for differences between group means of non-normally distributed variables. Partial correlation co-efficients (r) were used to examine the relationships between biochemical profile, nutrient intake data, food group intake data and dietary quality scores. All correlation analyses were controlled for potential confounding variables, including age, body mass index (BMI), energy intake and medication use. Participants with T2DM who were prescribed oral hypoglycaemic agents (OHAs) were excluded from such correlation analyses for glycaemic control. Results were considered statistically significant with values *P* < 0.05.

## Results

Data from a total of 111 Caucasian participants were collected for this cross-sectional study. Sixty-five participants had T2DM, 39 males and 26 females. Forty-six participants were in the control group, 16 males and 30 females. There was no significant difference between the T2DM group and the control group with regard to age or activity level due to strict study inclusion criteria.

### Subject characteristics

Subject characteristics are presented in Table 1. Thirty-nine percent of the study population (n = 43) were defined as overweight (BMI; 27.8 ± 1.2) (males = 28.0 ± 1.1, females = 27.6 ± 1.3) and a further 48% as obese (n = 53), (BMI; 34.8 ± 2.9) (males = 32.1 ± 2.5, females = 35.8 ± 5.1) [20]. The T2DM group had a significantly greater BMI than the control group (males = 28.5 ± 3.4, females = 27.1 ± 6.6, *P* < 0.001). Forty percent of participants with T2DM (n = 26) were overweight (BMI; 28.1 ± 1.1) (males = 28.2 ± 1.0, females = 28.0 ± 1.3) and a further 58% were obese (n = 38) (BMI; 35.7 ± 4.7) (males = 33.2 ± 2.6, females = 38.7 ± 4.9). This was greater than the control population where 37% were overweight (n = 17) (BMI 27.7 ± 1.2) (males = 27.9 ± 1.2, females = 27.3 ± 1.5) and 33% were obese (n = 15) (BMI; 33.9 ± 1.2) (males = 31.0 ± 1.3, females = 33.0 ± 2.5). There was also a significant difference in WC (*P* = 0.021) and WHR (*P* = 0.001) between the T2DM group (WC; males = 105.5 ± 9.0 cm, females = 111.5 ± 16.8 cm) (WHR; males = 0.9 ± 0.2, females = 1.0 ± 0.1) and the control group (WC; males = 102.4 ± 6.7 cm, females = 80.5 ± 0 cm) (WHR; males = 1.0 ± 0, females = 0.9 ± 0.1), with the T2DM group having the greatest measure within all parameters. The T2DM group had higher fasting plasma glucose, HbA1c and triacylglyceride levels, and a lower high density lipoprotein cholesterol level (all, *P* < 0.05). Sixty-two participants (n = 57 with T2DM and n = 5 control participants) were prescribed medications. Of the 57 participants with T2DM, 49 were taking OHAs, 45 were taking anti-hypertensive medications, 27 were prescribed statin medications. Of the control participants, 2 were prescribed anti-hypertensive medications and 5 were prescribed statin medication. The control participants were a representative sample of the national population in terms of BMI and habitual nutrient intake by comparison with the recently published national data [21].

**Table 1:** Mean (SD) subject characteristics of the T2DM and control participants

	**T2DM**			**Controls**			***P*value**
	**Mean**	**SD**	**N**	**Mean**	**SD**	**N**	
Age (y)	56	7.7	65	55	9.5	46	0.459
**Anthropometry**							
Height (m)	1.7	0.1	65	1.7	0.1	46	0.564
Weight (kg)	92.1	14.1	65	78.3	16.2	46	<0.001
BMI ^1^	32.5	5.3	65	27.6	4.1	46	<0.001
WC (cm)	107.4	12.2	54	101.2	8.4	17	0.021
WHR ^3^	0.95	0.2	64	0.93	0.1	45	0.001
% Adiposity^3^	32.6	5.4	65	29.3	4.1	46	0.003
**BMI category**^**a**^							
18.5-24.9 ^b^	1	-	-	14	-	-	-
25.0-29.9 ^b^	26	-	-	17	-	-	-
30.0-34.9 ^b^	20	-	-	13	-	-	-
35.0-39.9 ^b^	12	-	-	2	-	-	-
>40 ^b^	6	-	-	0	-	-	-
**Biochemical profile**							
FPG (mmol/L)^2^	7.8	1.8	61	4.6	0.6	42	<0.001
Insulin (mU/L)	13.5	8.5	51	-	-	-	-
HbA1c (%)^2^	6.8	0.9	61	5.5	0.4	33	<0.001
TC (mmol/L)^1^	4.4	0.9	60	5.2	1.2	43	<0.001
HDL-C (mmol/L)	1.3	0.2	52	1.6	0.5	43	0.002
LDL-C (mmol/L)	2.7	0.8	52	3.5	1.0	43	<0.001
TAG (mmol/L)^1^	1.8	0.7	60	1.3	0.6	42	<0.001

*T2DM* type 2 diabetes mellitus, *SD* standard deviation, *N* subject numbers, *BMI* body mass index, *WC* waist circumference, *WHR* waist-hip ratio, *FPG* fasting plasma glucose, *HbA1c* glycosylated haemoglobin, *TC* total cholesterol, *HDL-C* high density lipoprotein cholesterol, *LDL-C* low density lipoprotein cholesterol, *TAG* triacylglycerides.

* ^a^ World Health Organisation classification ^(20)^; ^b^ Expressed as frequency.

^1^Square root transformation; ^2^ Log transformation; ^3^ Non-parametric 2-tailed t-test analysis.

All other p-values obtained by independent 2-tailed t-test analyses between T2DM group and control group.

Subject numbers may vary due to missing data.

Statistically significant (*P* < 0.05).

### Nutrient intake analyses

Table 2 presents mean daily nutrient intake of the sample population. Application of Goldberg cut-off values for EI_rep_:BMR_est_ found that 74 participants under-reported energy intake. The level of under-reporting of energy intake was found to be equally distributed between both groups. No statistically significant differences were found in mean macronutrient intakes between the two groups and when micronutrient intake was assessed, it was found that those with T2DM consumed significantly less Vitamin D (4.2 ± 5.8 μg/10 MJ) than the control group (8.1 ± 6.7 μg/10 MJ) (*P* < 0.001). In addition, the T2DM group scored lower than the control group when Micronutrient Adequacy was assessed (T2DM; 1.6 ± 1.4, control; 2.3 ± 1.4, *P* = 0.009).

**Table 2:** Mean (SD) daily nutrient intake of T2DM and control groups

	**T2DM**			**Controls**			***P*value**
	**Mean**	**SD**	**N**	**Mean**	**SD**	**N**	
Energy (MJ/day)	7.7	2.1	65	7.8	2.3	46	0.519
Protein (g)	81.3	22.8	65	83.4	26.6	46	0.632
% en protein^1^	18.0	3.2	65	18.4	5.0	46	0.905
CHO (g)^1^	206.9	67.7	65	199.8	71.3	46	0.128
% en CHO	42.8	7.7	65	40.0	7.8	46	0.183
Total sugars (g)^1^	73.1	31.0	65	78.4	33.2	46	0.737
% en total sugars^2^	15.2	5.0	65	15.8	5.4	46	0.326
NMES (g)^2^	29.1	23.4	65	32.6	22.4	46	0.741
Fibre (AOAC) (g)^1^	17.9	5.5	65	18.7	6.0	46	0.861
Total fat (g)	72.5	26.7	65	73.2	26.1	46	0.525
% en total fat	35.2	8.0	65	34.9	6.0	46	0.824
SFA (g)	25.6	10.4	65	25.9	9.8	46	0.498
% en SFA	12.5	3.9	65	12.3	2.8	46	0.622
MUFA (g)	23.5	9.2	65	24.1	9.9	45	0.592
% en MUFA	11.4	3.0	65	11.4	2.8	45	0.824
PUFA (g)	13.2	6.1	65	11.6	5.8	45	0.084
% en PUFA	6.3	2.2	65	5.6	2.0	45	0.066
MUFA:SFA^1^	0.9	0.2	65	0.9	0.2	45	0.849
PUFA:SFA	0.5	0.2	65	0.4	0.2	45	0.090
Cholesterol (mg)^1^	252	117	65	283	153	46	0.925
Trans fats (g)^2^	1.1	1.0	65	1.1	1.0	45	0.912
% en trans fats^3^	0.5	0.4	65	0.5	0.4	45	0.796
Alcohol (g)^1^	11.9	14.6	65	17.9	18.4	46	0.912
% en alcohol^1^	4.1	5.0	65	6.3	6.8	46	0.964
Sodium (mg/10 MJ)	3745.2	794.0	65	3558.8	1133.7	46	0.137
Vitamin C (mg/10 MJ)^1^	209.4	434.8	65	139.9	75.5	46	0.184
Vitamin E (mg/10 MJ )^1^	10.6	4.4	65	11.7	5.3	46	0.264
Vitamin D (μg/10 MJ)^1^	4.2	5.8	65	8.1	6.7	46	0.000
Carotene (μg/10 MJ)^3^	3484.2	2357.3	65	5066.4	6314.6	45	0.253
Folate (μg/10 MJ)^3^	367.3	189.3	65	501.6	994.3	46	0.756
Potassium (mg/10 MJ)	4022.1	856.6	65	4185.1	851.1	46	0.324
Magnesium (mg/10 MJ)^1^	371.5	70.0	65	398.4	100.6	46	0.133
Zinc (mg/10 MJ)^3^	12.3	2.95	65	13.8	5.6	46	0.148
Micronutrient Adequacy Score (range 0–8)	1.6	1.4	65	2.3	1.4	46	0.009

*T2DM* type 2 diabetes mellitus, *SD* standard deviation, *N* subject numbers, *% en* percentage total energy, *CHO* carbohydrate, *NMES* non-milk extrinsic sugars, *SFA* saturated fatty acids, *MUFA* mono-unsaturated fatty acids, *PUFA* poly-unsaturated fatty acids.

^1^ Log transformation; ^2^ Square root transformation; ^3^ Non-parametric 2-tailed t-test analysis. All other p-values obtained by independent 2-tailed t-test analyses between T2DM group and control group.

Subject numbers may vary due to missing data.

Statistically significant (*P* < 0.05).

### Food group analyses

Due to the nature of recruitment and analysis performed, food group data was not available for all participants and has resulted in some missing values. In total, 31 subjects had missing values for some parameters. Mean daily intake of 19 food groups, measured by weight, is presented in Table 3. The top 5 most commonly consumed food groups of the T2DM group were, in descending order of intake, alcoholic drinks, fruit, meat and meat products, vegetables, and bread. For the control group, the top 5 foods consumed were alcoholic drinks, vegetables, fruit, potatoes and meat and meat products. Participants with T2DM were found to consume significantly more bread (*P* = 0.019), fats and oils (*P* = 0.021) and processed meats (*P* = 0.026) and significantly less vegetables (*P* = 0.046) than the control group.

**Table 3:** Mean (SD) daily food group intake of T2DM and control groups

	**T2DM**			**Controls**			***P*value**
	**Mean**	**SD**	**N**	**Mean**	**SD**	**N**	
***Food group***							
Bread (g)^3^	112.9	69.9	47	74.2	48.6	45	0.003
Breakfast cereals (g)^2^	42.1	43.8	47	53.8	52.7	45	0.327
Potatoes (g)^1^	96.8	59.8	47	108.6	80.5	45	0.283
Rice/pasta/other (g)^3^	50.3	56.0	47	76.5	81.2	45	0.141
Wholegrains (g)^2^	42.1	41.7	39	34.6	39.0	41	0.161
Legumes (g)^2^	8.2	20.9	39	9.3	16.2	41	0.280
Vegetables (g)	129.3	58.7	47	155.8	94.3	45	0.046
Fruit (g)	162.0	95.3	47	151.4	118.9	45	0.088
Nuts (g)^2^	5.9	10.7	39	6.5	19.7	41	0.089
Seeds (g)^2^	2.1	6.1	39	9.0	21.2	41	0.240
Meat & meat products (g)^2^	135.8	67.8	47	108.2	78.4	45	0.748
Fish (g)^2^	19.6	31.5	47	57.3	60.0	45	0.933
Red meat (g)^3^	62.3	48.7	39	51.1	42.3	41	0.317
White meat (g)^3^	41.8	34.0	39	41.2	62.3	41	0.136
Processed meats (g)^3^	35.2	29.0	39	24.1	34.0	41	0.026
Milk / cream (g)^2^	198.54	134.9	49	106.98	97.62	45	0.000
Cheese (g)^2^	13.74	17.31	49	17.93	23.08	45	0.913
Yogurts (g)^2^	40.76	50.51	41	35.36	45.51	41	0.241
Ice-cream (g)^2^	4.87	11.82	41	3.10	8.44	41	0.841
Biscuits & cakes (g)^2^	47.4	50.4	47	43.6	44.4	45	0.725
Sugar & confectionary (g)^2^	19.5	32.9	47	31.0	61.6	45	0.181
Fats & oils (g)^3^	15.6	10.7	47	11.8	10.4	45	0.048
Alcoholic drinks (g)^2^	251.2	425.0	47	298.4	392.0	45	0.063

*T2DM* type 2 diabetes mellitus, *SD* standard deviation, *N* subject numbers.

^1^Square root transformation; ^2^ Log transformation; ^3^ Non-parametric t-test analysis. All other p-values obtained by independent 2-tailed t-test analyses between T2DM group and control group.

Subject numbers may vary due to missing data.

Statistically significant (*P* < 0.05).

### Dietary quality analyses

Table 4 presents the mean dietary quality scores for the T2DM group and control group as per the Mediterranean Diet Score, the Alternate Mediterranean Diet Score, the Healthy Diet Indicator and the Alternate Healthy Eating Index. The T2DM group were found to have a significantly lower (*P* < 0.001) Mediterranean Diet Score when compared to the control group (T2DM; 3.4 ± 1.3, control; 4.8 ± 1.8). A similar result was also found when the Alternate Mediterranean Diet Score was applied (T2DM; 3.3 ± 1.5, control; 4.2 ± 1.8, *P* = 0.022). The T2DM group were also found to have a significantly lower (*P* = 0.001) Healthy Diet Indicator score than the control group (T2DM; 2.6 ± 2.3, control; 3.3 ± 1.1), indicating poorer quality of dietary intake in the T2DM group. No significant difference was noted in Alternate Healthy Eating Index Scores between the T2DM and control groups.

**Table 4:** Mean (SD) dietary quality index scores for T2DM and control groups

	**T2DM**			**Controls**			***P*value**
	**Mean**	**SD**	**N**	**Mean**	**SD**	**N**	
***Dietary quality index***							
Mediterranean diet score^1^	3.4	1.3	39	4.8	1.8	41	<0.001
Alternate mediterranean diet score^1^	3.3	1.5	39	4.2	1.8	41	0.022
Healthy diet indicator^1^	2.6	2.3	39	3.3	1.1	41	0.001
Alternate healthy eating index	40.2	10.8	39	41.9	11.7	41	0.513

*T2DM* type 2 diabetes mellitus, *SD* standard deviation, *N* subject numbers.

^1^ Non-parametric 2-sided t-test analysis. All other p-values obtained by independent 2-sided t-test analyses between T2DM group and control group.

Subject numbers may vary due to missing data.

Statistically significant (*P* < 0.05).

### Correlation analyses

Spearman rho correlation analyses found that a number of chosen DQIs were significantly correlated to each other. The Mediterranean Diet Score had a significant positive correlation to the Alternate Mediterranean Diet Score (r = +0.7, *P* < 0.001), the Alternate Healthy Eating Index (r = +0.4, *P* < 0.001) and the Healthy Diet Indicator (r = +0.3, *P* = 0.001). The Alternate Mediterranean Diet Score was also found to be positively correlated to the Alternate Healthy Eating Index (r = +0.6, *P* < 0.001) and the Healthy Diet Indicator (r = +0.3, *P* = 0.007). No other significant correlations were observed.

Partial correlation analyses were also carried out between nutrient, food group, anthropometrical and biochemical data. Further significant associations observed are presented in Table 5. Partial correlation analyses were also carried out between nutrient, food group, anthropometrical and biochemical data and further significant associations observed are presented in Table 5. Table 6 outlines the scoring system for each dietary quality index and the relevant health outcomes which were found to be associated with each index in the published literature.

**Table 5:** Significant partial correlations of dietary intake, anthropometry and biochemical parameters for total sample population

**Variable 1**	**Variable 2**	**r value**	***P*value**
Weight (kg)	FPG	+0.37	<0.001
	HbA1c	+0.27	0.009
	Insulin^1^	+0.34	0.032
	TAG	+0.45	<0.001
BMI	FPG	+0.43	<0.001
	HbA1c	+0.27	0.006
	Insulin^1^	+0.48	<0.001
	TAG	+0.43	<0.001
WC (cm)	Insulin^1^	+0.53	<0.001
	TAG	+0.35	0.014
	Fibre	−0.28	0.018
Mediterranean diet score	BMI	−0.27	0.042
	FPG	−0.36	0.001
	HbA1c	−0.26	0.024
Alternate mediterranean diet score	FPG	−0.27	0.021
Healthy diet indicator	FPG	−0.25	0.027

*FPG* fasting plasma glucose, *HbA1c* glycosylated haemoglobin, *TAG* triacylglycerides, *BMI* body mass index, *WC* waist circumference.

^1^ T2DM group only.

Partial correlation coefficients (r) controlled for covariates; Participants taking statin medications excluded from lipid analysis; Participants taking oral hypoglycaemic agents excluded from glycaemic profile analysis; All analyses controlled for age; Dietary intake analysis control for energy intake. No significant partial correlations observed in relation to alternate healthy eating index.

Statistically significant (*P* < 0.05).

**Table 6:** Summary of dietary quality indices

**Dietary quality index**	**Author(s)**	**Score**	**Measure**	**Associations**
**Mediterranean diet score**	Trichopoulou *et al.,* 1995	Range: 0–9	Adherence to a traditional Mediterranean dietary pattern	● Inversely associated with overall mortality [9]
				● Significant reduction in total mortality [11]
				● Inversely associated with cancer disease risk [22-24]
**Alternate mediterraneandiet score**	Fung *et al.,* 2005	Range: 0–9	Adherence to a Mediterranean dietary pattern. Adapted score to give greater focus on within food group quality	● Inverse association with inflammatory biomarkers [12]
				● Lower incident of mortality from coronary heart disease and stroke [25]

				● Significant inverse association with BMI and obesity [26]
**Alternate healthy eating index**	McCullough *et al.,* 2002	Range: 2.5–87.5	Adherence to USA dietary guidelines and the USA My Food Pyramid. Adapted score to give greater focus on within food group quality	● Significant reduction in overall chronic disease risk, with greater strength in prediction of chronic disease risk when compared to the original Healthy Eating Index [27]
				● Inverse association with inflammatory biomarkers [12]
				● Reduction in overall disease risk and risk of premature mortality from coronary vascular disease [28]
**Healthy diet indicator**	Huijbregts *et al.,* 1997	Range: 0–9	Adherence to WHO 1990 dietary recommendations for the prevention of chronic disease	● Significantly inverse association with 20 year all-cause mortality in a multi-cultural population [13]
				● Significantly correlated with nutritional adequacy (MAR) [29]

*BMI* Body mass index, *USA* United States of America, *MAR* mean adequacy ratio.

## Discussion

This study found that the overall quality of dietary intake consumed by a sample of individuals with T2DM was less than that of a healthy control population when measured using the Mediterranean Diet Score, the Alternate Mediterranean Diet Score and the Healthy Diet Indicator. Conversely, when individual macronutrient intakes were assessed, no differences were observed between those with and without T2DM. Given that the design of the current study is of a cross-sectional case–control nature, a cause and effect relationship between dietary quality and incidence of T2DM cannot be determined.

Lifestyle is considered a cornerstone both in the treatment and management of T2DM [30,31]. The results of the current study show that individuals with T2DM consumed a diet significantly less like the Mediterranean dietary pattern compared to a non-T2DM population, as assessed by the Mediterranean Diet Score and Alternate Mediterranean Diet Score. In addition, there was a significant inverse association between the Mediterranean diet score and BMI as well as fasting plasma glucose and fasting plasma insulin, recognised as parameters of glycaemic control. Recent evidence from both observational analyses and intervention studies suggest that adherence to a specific dietary pattern that promotes metabolic health may be more beneficial than adherence to individual nutrient based recommendations [32]. The Mediterranean dietary pattern is characterised by high intakes of fibre, lean meats and fruit and vegetables [9,11]. Concern was initially raised as to the relatively high fat content of the Mediterranean diet, which can have up to 40% total energy derived from fat, and the effects that this could mediate on body mass and metabolic health. However, several epidemiological studies have shown that the Mediterranean dietary pattern is inversely associated with weight gain, BMI and T2DM risk [26,33,34]. Furthermore, evidence suggests that the Mediterranean dietary pattern has beneficial effects on lipid and glycaemic profiles [12,35-37].

The current study showed that those individuals with T2DM had a lower score than the control group when dietary pattern was assessed using the Healthy Diet Indicator (HDI). Whereas several epidemiological studies and systematic reviews have examined the relationship between the Mediterranean dietary pattern and T2DM risk [7,32,33], to the best of our knowledge the current study is the first to examine dietary intake using the HDI in a sample of individuals with T2DM. The HDI is based on the WHO guidelines for diet and nutrition in the prevention of chronic disease and whilst the current study design prevents cause and effect inferences to be drawn, it is interesting that the T2DM cohort scored significantly lower than the control participant using this dietary pattern score. Conversely, no significant difference was found in dietary quality scores between the T2DM and control groups when measured using the Alternate Healthy Eating Index. While speculative, this result may be due to the fact that while the other DQIs use a predefined cut off point in the allocation of scores, the Alternate Healthy Eating Index allocates scores for a wider range of intakes.

Individuals with T2DM also scored significantly lower when micronutrient intake was assessed using the Micronutrient Adequacy Score, a result that highlights that although micronutrient adequacy was suboptimal in both groups, individuals with T2DM consumed a more nutrient dilute diet than their insulin sensitive counterparts. When the intake of nine individual micronutrients was assessed, individuals with T2DM consumed significantly less Vitamin D than the control group. Emerging evidence suggests that supplementing vitamin D in individuals with impaired glucose tolerance improves insulin sensitivity, however results from those with T2DM have been inconsistent [38,39].

Much research has been carried out on the effects of different dietary patterns on body composition, metabolic health and disease risk. A Western dietary pattern with characteristic consumption of high levels of saturated fatty acids, red and processed meats, confectionery and refined grains has been associated with increased T2DM risk and deterioration of metabolic health, independent of weight status [2,40,41]. In the current study, food group analyses found that the T2DM group had a high consumption from the fruit and vegetable food groups yet failed to reach the recommended intake of 400 g of fruit and vegetables per day [31]. The third highest food group consumption of the T2DM group came from meat and meat products. Total cereal grain consumption was notably higher than consumption of wholegrain cereals alone for the T2DM group. This suggests a greater consumption of refined cereal grains within this group. The T2DM group also consumed significantly more fats and oils, processed meats and bread than the control group. These findings represent features of a Western dietary pattern. Conversely, consumption of a prudent dietary pattern, which includes intake of fruit and vegetables, lean meats and wholegrains, characteristic components of a Mediterranean dietary pattern, has been found in other studies to be protective to metabolic health [2,42].

Evidence suggests that even short term hyperglycaemia results in increased vascular intracellular adhesion molecules in individuals with T2DM, a risk factor for atherosclerosis [43,44]. This supports a need for greater adherence to a dietary pattern that promotes increased amounts of fibre containing foods in order to regulate the hyperglycaemic response [45,46]. Fibre independently affects metabolic health. The current analysis found that total fibre intake had a significant negative association with waist circumference. Similar results have also been found in previous analyses [47,48]. Given the substantial evidence to support WC as an indicator of cardiometabolic disease risk [49], the current results suggest that fibre may play a role in the attenuation of obesity induced metabolic dysregulation.

Excess adiposity is associated with increased morbidity and mortality [50]. Thirty-nine percent of the total population in this study were overweight. This is consistent with recent findings which concluded that 37% of a representative sample of the Irish population were overweight [21]. At group level, the control group have a similar mean BMI, 27.6, when compared to a representative sample of the Irish population at 27.5. However, the T2DM group was found to have a BMI of 32.5 which is significantly greater than the control population. Given the known association between the Mediterranean Diet Score and BMI and weight status [26,34], it must be noted that such differences in BMI between the two groups may have potentially contributed to the T2DM group having a significantly lower dietary quality score than the control group.

The strengths of the current study were that at group level there was no significant difference in age or activity level between the participants with T2DM and the control group, and that control participants were a representative sample of the national population in terms of BMI and habitual nutrient intake by comparison with the recently published national data [21]. There are however, limitations associated with the current study. The first is the fact that validation of reported dietary energy intake data using the Goldberg method [51] revealed significant misreporting in this sample of Irish adults. Based on estimates of energy expenditure, seventy-four of the 111 subjects (66.6%) in this study were found to under-report their dietary intake, which is likely to explain the relatively low mean energy intake observed. However, the mean level of under-reporting was similar in both groups, suggesting that while mean nutrient intakes may be under-estimated in this cohort, any differences or lack thereof, detected between the two groups, remain. Studies suggest that the mis-reporting of dietary energy is a serious problem in any study of dietary intake [52]. Moreover, obesity affects both the quantity and quality of reported dietary energy intake data [53,54]. As 86% of the participants of this current study were overweight or obese (98% T2DM and 70% control) this will without doubt have contributed to the high level of energy under-reporting observed. Furthermore, a high level of under-reporting of energy intake has also been noted in individuals with T2DM [55,56]. The second limitation is that although the two groups were similar in terms of age and activity level, a significant difference was found in BMI between the two groups. Thirdly, it must also be noted that some participants in the T2DM group may have previously received dietary advice from a healthcare professional which may have affected habitual dietary intake in this group. The fourth limitation is that there was no gender balance between the adults with T2DM and the controls. There were 39 male and 26 female participants with T2DM yet there were only 16 males and 30 females in the control group. Another limitation is that the cases and controls were not matched socio-economically. Whilst they were all recruited from the same catchment area in Dublin, Ireland, no objective measure of socio-economic status was used. A final limitation is that this study also has a cross-sectional design with a relatively small sample size allowing only observation of any associations between nutrient intake, dietary quality and metabolic health in a sample of individuals with T2DM. Further confirmation of these findings will require large scale randomized controlled trials.

## Conclusions

In conclusion, the current cross-sectional study found that when compared to a control population, participants with T2DM consumed a diet of lower overall quality, despite the fact that no differences in individual macronutrient intake were detected. When dietary intake was assessed using a series of validated dietary quality indices, the T2DM group consumed a dietary pattern that is more representative of a typical westernised dietary pattern than the control population. These findings suggest that using dietary patterns to model dietary recommendations may be more beneficial than nutrient based approaches.

## Competing interests

The authors declare that they have no competing interests.

## Authors’ contributions

AEM, AMM and FEL conceived and designed the research, analysed and interpreted the data and drafted the manuscript. EOC, CK, OME, ME and DOS were responsible for participant recruitment and data collection and DOS was the study clinician. All authors read and approved the final manuscript.

## Authors’ information

Alison E Murray and Aoibheann M McMorrow are joint first authors.
